# Seroprevalence of SARS-CoV-2 Among Pediatric Healthcare Workers in Spain

**DOI:** 10.3389/fped.2020.00547

**Published:** 2020-09-11

**Authors:** Ana Dacosta-Urbieta, Irene Rivero-Calle, Jacobo Pardo-Seco, Lorenzo Redondo-Collazo, Antonio Salas, Jose Gómez-Rial, Federico Martinón-Torres

**Affiliations:** ^1^Grupo de Investigación en Genética, Vacunas, Infecciones y Pediatría (GENVIP), Instituto de Investigación Sanitaria de Santiago (IDIS) and Hospital Clínico Universitario and Universidade de Santiago de Compostela (Servicio Gallego de Salud), Galicia, Spain; ^2^Translational Pediatrics and Infectious Diseases, Department of Pediatrics, Hospital Clínico Universitario de Santiago de Compostela, Galicia, Spain; ^3^Unidade de Xenética, Instituto de Ciencias Forenses (INCIFOR), Facultade de Medicina, Universidade de Santiago de Compostela, and GenPoB Research Group, Instituto de Investigaciones Sanitarias (IDIS), Hospital Clínico Universitario de Santiago (Servicio Gallego de Salud), Galicia, Spain; ^4^Laboratorio de Inmunología, Servicio de Análisis Clínicos, Hospital Clínico Universitario Santiago de Compostela (Servicio Gallego de Salud), Galicia, Spain

**Keywords:** seroprevalance, COVID-19, healthcare worker (HCW), SARS-CoV-2, rapid test for COVID-19

## Abstract

Spain is one of the countries most severely affected by the SARS-CoV-2 pandemic, with almost 190,000 cases as of April 18, 2020. As healthcare workers (HCW) are one of the groups hardest hit by the infection, it is important to know the seroprevalence of antibodies against SARS-CoV-2 in pediatric departments. We performed 175 immunoglobulin (Ig)M and IgG immunochromatographic rapid tests in the personnel working at the Pediatric Department of the Hospital Clínico Universitario of Santiago de Compostela (Spain), including pediatricians, residents, nurses, and other staff, on days 31–33 since the lockdown started. Seven out of the 175 tests were positive, including four for IgM and three for IgG, leading to a seroprevalence of 4.0% (95% CI: 1.1–6.9%). Only one of them had symptoms at the time of testing (sore throat). All seropositive cases yielded negative RT-PCR of the upper and lower respiratory tract. This is the first SARS-CoV-2 serological survey among HCWs reported in Spain. Notwithstanding the test limitations, our results reveal that personal protection policy and lockdown measures have been effective to limit population exposure. The low seroprevalence rate poses a significant challenge for the next strategic steps of pandemic control.

## Introduction

Spain is one of the countries with the highest number of cases of SARS-CoV-2 reported worldwide, with almost 190,000 cases confirmed as of April 18, 2020 (1). The first case in Spain was recorded on January 31, and the Spanish government started the lockdown on March 14, when the country had 7,753 active cases. In Galicia (northwest Spain), the first case was recorded on the March 4, and at the moment of the lockdown, there were 195 confirmed cases. In the last 2 weeks, the cumulative incidence of SARS-CoV-2 is 149.61 cases/100,000 inhabitants in Spain, and 103.5 cases/100,000 inhabitants (1) in Galicia. To date, we have identified 19 pediatric cases in our hospital, and only one of them was admitted to hospital for reasons unrelated to the infection. We aimed to assess the seroprevalence rate of SARS-CoV-2 among healthcare workers (HCWs) of the Pediatric Department of the Hospital Clínico Universitario de Santiago de Compostela, the reference hospital in Galicia.

## Methods

We performed a sero-epidemiological survey on days +31, +32, and +33 after lockdown started, including all HCWs of the Pediatric Department of the Hospital Clínico Universitario de Santiago de Compostela, namely, pediatricians, resident doctors, nurses, and administrative staff. Emergency Department and the Pediatric Intensive Care Unit were considered the most exposed areas.

We tested immunoglobulin (Ig)M and IgG against SARS-CoV-2 by an immunochromatographic rapid method (Virusee® by Genobio Pharmaceutical, Shanghai, China) using 10-μl finger-prick/capillary blood. Sensitivity and specificity rates specified by the manufacturer were 96.0% and 96.8% for IgG and 94.6% and 96.8% for IgM detection, respectively. The rapid test used provides the result in 10 min. Epidemiological, clinical, and laboratory data were collected in all cases.

## Results

The test was performed on 175 HCWs. Most of them were pediatric consultants (32.6%; *n* = 57), followed by nurses and nurses' aides (47.4%; *n* = 83), pediatrics residents (13.1%; *n* = 23), and others (6.9; *n* = 12). In addition, 18.3% (*n* = 32) of the workers were ≥55 years old, and 12.6% (*n* = 22) had preexisting comorbid conditions such as asthma (4.0%; *n* = 7), high blood pressure (1.7%; *n* = 3), or obesity (1.7%; *n* = 3). Moreover, 22.5% of the workers had a known exposure to SARS-CoV-2-positive patients. When asked if they recalled any symptoms suggestive of SARS-CoV-2 in the last 2 months, 53% of the workers declared none.

Seven workers yielded positive test results, three of them were IgG positive and four of them were IgM positive, representing a total seroprevalence of 4.0% of the cohort (95% CI: 1.1–6.9%) (Table 1). The subjects who tested positive for IgM worked in the most exposed areas were: two medical residents and one consultant, two nurses, and two nurse aides. None of the workers who tested positive for IgM presented symptoms at the time of the test. Of the subjects who tested positive for IgG, only one of them recalled symptoms suggestive of coronavirus infection (cough and sore throat), and these persisted at the time of testing. In the positive cases, RT-PCR was performed on the lower (oropharyngeal swab) and upper respiratory tracts (nasal swab), all with negative results.

**Table 1:** Characteristics of the seropositive subjects.

	**Subject 1**	**Subject 2**	**Subject 3**	**Subject 4**	**Subject 5**	**Subject 6**	**Subject 7**
Place of work	PW	PICU	PC	NICU	NICU	PW	PW
Staff category	NA	Nurse	Pediatrician	Nurse	RP	RP	NA
Age (years)	55–64	35–44	55–64	35–44	25–34	25–34	35–44
Exposure	No	No	Yes	No	Yes	No	No
House contacts	Yes	Yes	Yes	Yes	Yes	No	Yes
Pets at home	No	No	–	No	No	No	No
Comorbidities	No	No	–	Yes	Yes	No	No
Symptoms	No	No	No	No	No	No	Yes
Test result	IgM+/RT-PCR–	IgM+/RT-PCR–	IgM+/RT-PCR–	IgM+/RT-PCR–	IgG+/RT-PCR–	IgG+/RT-PCR–	IgG+/RT-PCR–
Treatment received	No	No	No	No	No	No	No

*PW, pediatric ward; PC, primary care; RP, pediatrics resident; NA, nurse aide; PCR–, RT-PCR negative; Exposure, exposure to SARS-CoV-2-positive patients; Symptoms, symptoms suggestive of SARS-CoV-2; Ig, immunoglobulin; PICU, pediatric intensive care unit; NICU, neonatal intensive care unit*.

## Discussion

To date, there are few data regarding serological responses to SARS-CoV-2 in infected patients (2) and virtually no data on serological responses to SARS-CoV-2 in asymptomatic exposure. Rapid tests using serology have the advantage of delivering quick results and allowing the testing of asymptomatic people reliably (3); the technique is easier and quicker than RT-PCR in respiratory samples. True sensitivity and specificity can vary depending on the commercial test, and this fact warrants the need for further studies. Despite the limitations of the rapid test applied, the limited number of subjects analyzed, and the lack of confirmatory alternative serological assays, the present study indicates a low exposure to SARS-CoV-2 among pediatric HCWs in Spain. There is only one other serologic study published in Spain (4) where the total number of participants with evidence of past or current infection (by PCR and/or serology) was 11.2% (65/578); however, these results come from a large referral hospital in Barcelona, Spain, one of the regions with the highest burden of disease of coronavirus disease 2019 (COVID-19) in Spain.

Although our results must be interpreted with caution since it is possible that some of the HCWs with IgG positivity could have acquired the infection outside the hospital, the low seroprevalence against SARS-CoV-2 among pediatric HCWs points to the success of personal protection and lockdown policies together with a low nosocomial risk of infection, while highlighting the challenge for the next stages of SARS-CoV-2 pandemic management.

## Data Availability Statement

The original contributions presented in the study are included in the article/supplementary material, further inquiries can be directed to the corresponding author/s.

## Ethics Statement

The studies involving human participants were reviewed and approved by Comité de Ética de la investigación con medicamentos de Galicia (CEIm-G). Written informed consent for participation was not required for this study in accordance with the national legislation and the institutional requirements.

## Author Contributions

All authors listed have made a substantial, direct and intellectual contribution to the work, and approved it for publication.

## Conflict of Interest

The authors declare that the research was conducted in the absence of any commercial or financial relationships that could be construed as a potential conflict of interest.
